# The effect of endoscopic ultrasound on the precise selection of endoscopic treatment for submucosal tumors in the upper gastrointestinal tract

**DOI:** 10.1186/s12893-023-02164-7

**Published:** 2023-08-27

**Authors:** Jian-Hua Li, Shu-Min Qin, Tian-Wen Liu, Jun-Qian Chen, Ying-Ting Li

**Affiliations:** Department of Gastroenterology, The Second Affiliated Hospital of Guangzhou University of Chinese Medicine, Guangzhou, 510006 China

**Keywords:** Endoscopic ultrasound (EUS), Submucosal tumor (SMT), Endoscopic treatment

## Abstract

**Objective:**

To summarize and discuss the guiding role of endoscopic ultrasound (EUS) in selecting endoscopic treatments for submucosal tumors (SMTs) in the upper gastrointestinal tract.

**Methods:**

A retrospective investigation was conducted on 156 SMT patients who received endoscopic resection guided by EUS in the endoscopy center of the Second Affiliated Hospital of Guangzhou University of Chinese Medicine from May 2019 to September 2021. Next, the size, pathological type, and distribution of lesions were analyzed; the correlation of the tumor origin with distribution of lesions and selection of treatments was explored; and the consistency of preoperative EUS diagnosis and postoperative pathological diagnosis was summarized and analyzed.

**Results:**

The tumor diameters of the included SMT patients ranged from 0.3 to 4 cm, with a mean diameter of 0.95 cm; the lesions were mostly located in the esophagus, gastric fundus or fundic cardia and gastric body. As for the pathological types, liomyoma was the most common tumor in the esophagus, liomyoma and mesenchymoma were mainly located in the fundic cardia and gastric body, and heterotopic pancreas was mostly discovered in the gastric sinus. Among 38 esophageal SMT patients, some with lesions originating from muscularis mucosa and submucosa under EUS mainly underwent endoscopic submucosal dissection (ESD) and endoscope band ligation (EBL); while others with lesions originated from muscularis propria mainly received submucosal tunneling endoscopic resection (STER). Of 115 gastric SMT patients under EUS, some with lesion origins from the muscularis mucosa and submucosa mainly underwent endoscopic submucosal excavation (ESE), while others from muscularis propria mainly underwent ESE, ESD, and endoscopic full-thickness resection (EFTR). Besides, 3 duodenal SMT patients with lesion origins from submucosa and muscularis propria under EUS were given ESD and ESE, respectively. Additionally, 121 cases showed a consistency between the EUS diagnosis and the postoperative pathological nature, and the consistency rate was 84.6%.

**Conclusion:**

Clarifying the origin layer, size, growth pattern, and pathological nature of the lesion through preoperative EUS can guide the precise selection of endoscopic treatments, thereby ensuring a safe, effective, and complete surgical outcomes and reducing complications.

## Introduction

Submucosal tumor (SMT) is a broad term for tumors originating from tissues below the mucosal layer, including mucosa, submucosa and lamina propria [1]. Common SMT consists of mesenchymomas, liomyomas, lipomas, neurogenic tumors, fibromas, and hemangiomas [2]. Generally, SMT in the upper gastrointestinal tract mostly occurs in the esophagus and stomach [3]. The incidence of SMT has been rising steadily in recent years due to the focus on gastroscopy, with mesenchymoma being the most common pathological type [4]. Due to the presence of malignant potential, SMT may fail to be treated with minimally invasive surgery if it’s allowed to be grown freely. Therefore, endoscopists have long focused on how to perform a safe and effective endoscopic resection of SMT. Clinically, endoscopic treatment is mainly based on the location, size, symptoms, and histological type of SMT, as well as the own situation of patients. At present, the main endoscopic treatments for SMT in the upper gastrointestinal tract include endoscope band ligation (EBL), endoscopic submucosal dissection (ESD), endoscopic submucosal excavation (ESE), endoscopic full-thickness resection (EFTR), and submucosal tunneling endoscopic resection (STER) [2]. Notably, endoscopic ultrasound (EUS) can reveal information about the origin layer, growth pattern, size, and pathological nature of SMT [5, 6]. In this study, the patients with SMT in the upper gastrointestinal tract treated between May 2019 and September 2021 were analyzed. Collectively, under guidance of EUS, doctors can select surgical treatments more precisely, thereby ensuring the effect of endoscopic treatment and avoiding postoperative complications.

## Methods

### General information

The patients meeting inclusion and exclusion criteria were selected for data extraction. The inclusion criteria included patients ① over 18 years old and ② with SMT in the upper gastrointestinal tract who underwent endoscopic resection guided by EUS. The exclusion criteria included patients ① with incomplete case information or ②case information failing to be extracted. Finally, 156 patients admitted to our endoscopy center from May 2019 to September 2021 were included. The included patients were composed of 68 males and 88 females, the age ranged from 21 to 92 years, and the mean age was 53.93 years. The clinical data of patients were retrospectively analyzed and summarized. Besides, this study was approved by the Ethics Committee of the Second Affiliated Hospital of Guangzhou University of Chinese Medicine (Ethics No. ZE2023-033).

### Examination methods and the applied instruments

For lesions with the diameter shorter than 2 cm, the ultrasound endoscope mainframe was OLYMPUS EU-ME2 (Olympus Corporation, 7,016,223, Tokyo Metropolis, Japan), the microprobe driver was MAJ-935 (Olympus Corporation, SFDA(I)20,123,232,155, Tokyo Metropolis, Japan), and the probe was UM-3R (20 MHz) (Olympus Corporation, 7,173,855, Tokyo Metropolis, Japan). Besides, the PENTAX Medical ultrasound endoscope (HOYA Corporation, EE012155, Tokyo Metropolis, Japan) was employed for assessing lesions with the diameter over 2 cm, or that could not be displayed clearly by the scanning of the ultrasound small probe, or accompanied with extraluminal growth, with a frequency of 7.5 to 10 MHz. All scans were performed using the water-filling method.

### Preoperative preparation

All patients stopped taking anticoagulant and antiaggregation drugs 1 week before surgery and received routine blood and coagulation tests. Fasting and water prohibition were required at 8 h before surgery. All patients were informed of the risks and possible complications associated with endoscopic treatment and signed an informed consent form. All patients received endotracheal intubation for anesthesia and preoperative EUS for clarifying the origin layer, growth pattern, and size of the lesion. Notably, tumors > 2 cm required abdominal CT to assess association of the tumour with the surrounding organs and blood vessels. Additionally, CO_2_ gas was used intraoperatively. According to the EUS results, the appropriate endoscopic treatment procedure was adopted.

### Treatment procedures

#### Endoscope band ligation (EBL)

Adoption criteria: SMT (1) originating from muscularis mucosa and submucosa of the esophagus, with intraluminal growth and a diameter ≤ 0.8 cm; (2) derived from muscularis mucosa, submucosa and propria of the stomach, with intracavitary growth and a diameter ≤ 0.8 cm.

**Fig. 1:**
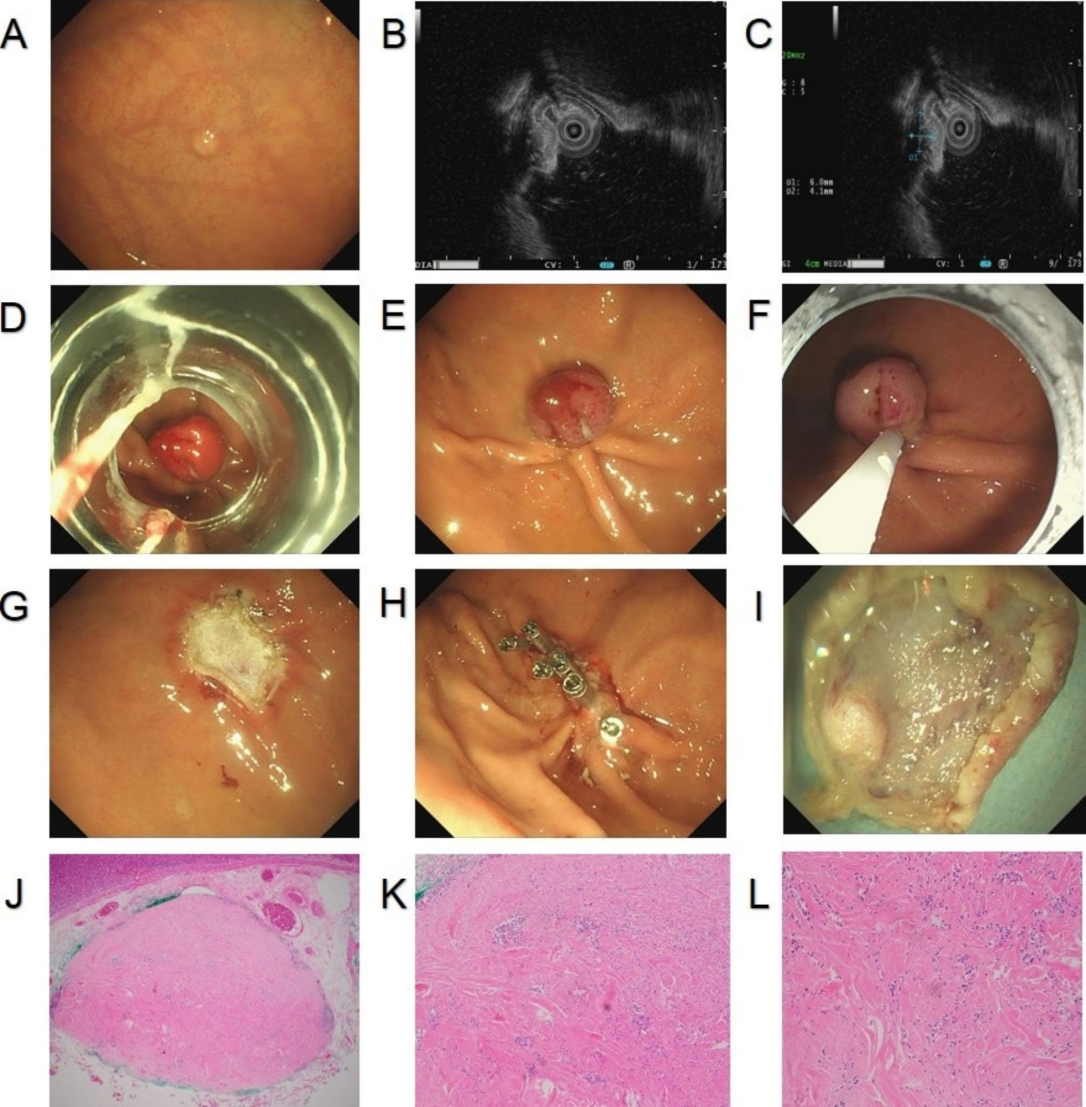
**A**. Gastric submucosal tumors under gastroscopy; **B**. hyperechoic masses originating from submucosa and with intraluminal growth under EUS; **C**. Size of a tumor section under EUS: 0.60 cm*0.41 cm; **D**. Tumor ligation by the ligation device; **E**. Rubber ring ligation of the tumor; **F**. A high-frequency electroresection for the tumor caught by the snare; **G**. Trauma after electroresection; **H**. Closure of trauma by titanium clip; **I**. The completely resected tumor; **J-L**. Histopathological diagram of calcified fibroma originating from submucosa. EUS, endoscopic ultrasound

Procedure: After installing the ligation device at the front of the gastroscope, the lesion was sucked into the transparent cap through negative pressure suction. Immediately a rubber ring was released to ligate the lesion. Following that, the ligation device was removed, and the lesion was caught with a snare under the rubber ring. Finally, the lesion was subjected to high-frequency electrocoagulation and electroresection (Fig. 1A-L).

#### Endoscopic submucosal dissection (ESD)

Adoption criteria: (1) SMT originating from muscularis mucosa and submucosa of the esophagus, with a diameter > 0.8 cm; (2) SMT derived from muscularis mucosa and submucosa of the stomach and duodenum, with intracavitary growth.

**Fig. 2:**
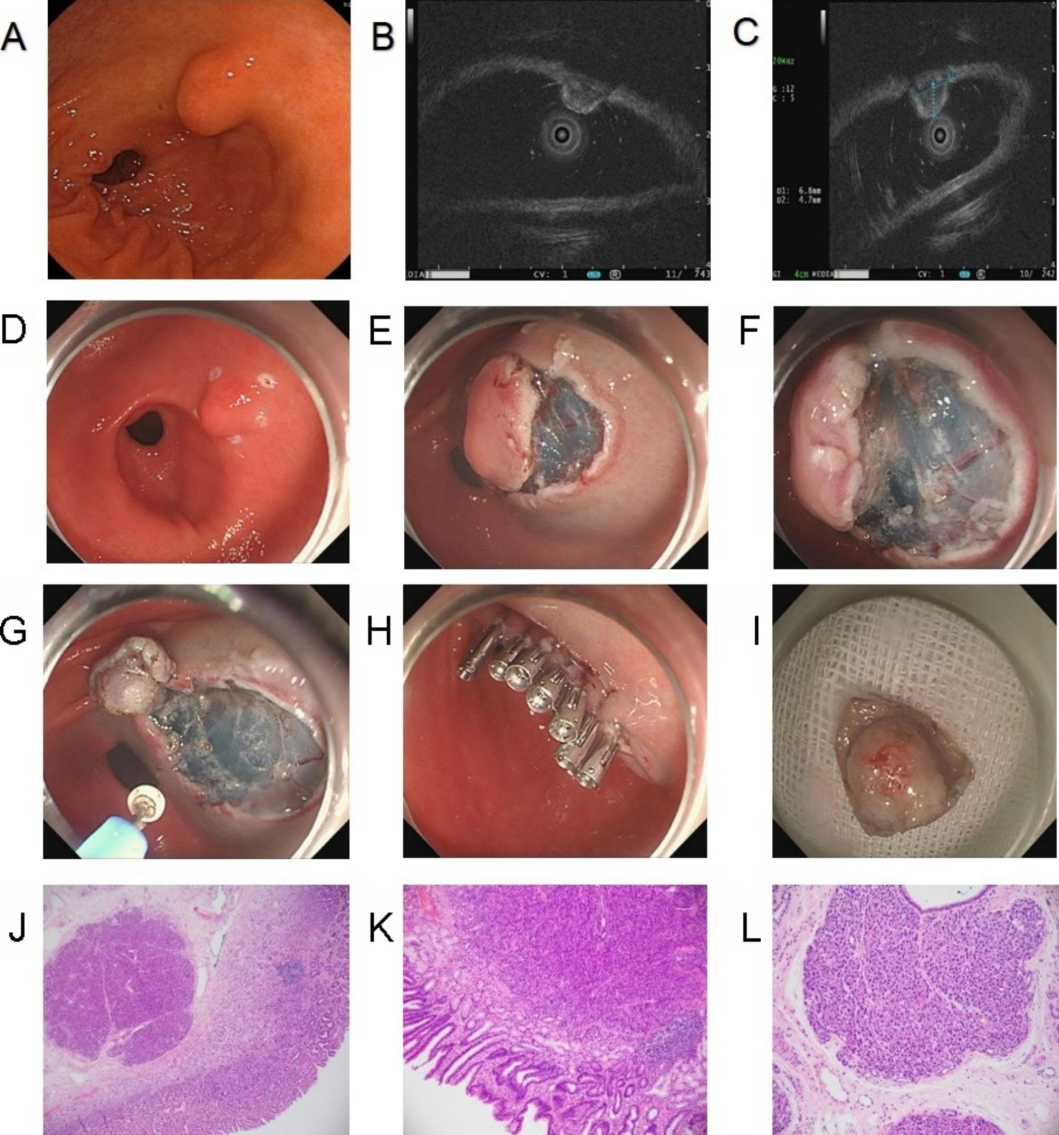
**A**. Submucosal tumor at gastric sinus under gastroscopy; **B**. Mixed echo masses originating from submucosa under EUS; **C**. Tumor section size under EUS: 0.68 cm*0.47 cm; **D**. Marking at the edge of the tumor; **E**. Circumferential incision along the marks after submucosal injection; **F**. Exposure and debridement of tumor; **G**. Trauma after the debridement; **H**. Closure of trauma by titanium clip; **I**. Complete debridement of tumor; **J-L**. Histopathological diagram of heterotopic pancreas originating from submucosa. EUS, endoscopic ultrasound

Procedure: Marks were made around the lesion, followed by a submucosal injection of the mixture (glycerol fructose + sodium glacial + indocyanine + epinephrine). Subsequently, C-shape incision or circumferential incision of the muscularis mucosa and submucosa was performed along the marks using an electric knife. After separation of the submucosa and exposure of the lesion, the lesion was completely extracted along the tumor edge and base layer by layer (Fig. 2A-L).

#### Endoscopic submucosal excavation (ESE)

Adoption criteria: SMT originating from the muscularis propria of the stomach and duodenum.

**Fig. 3:**
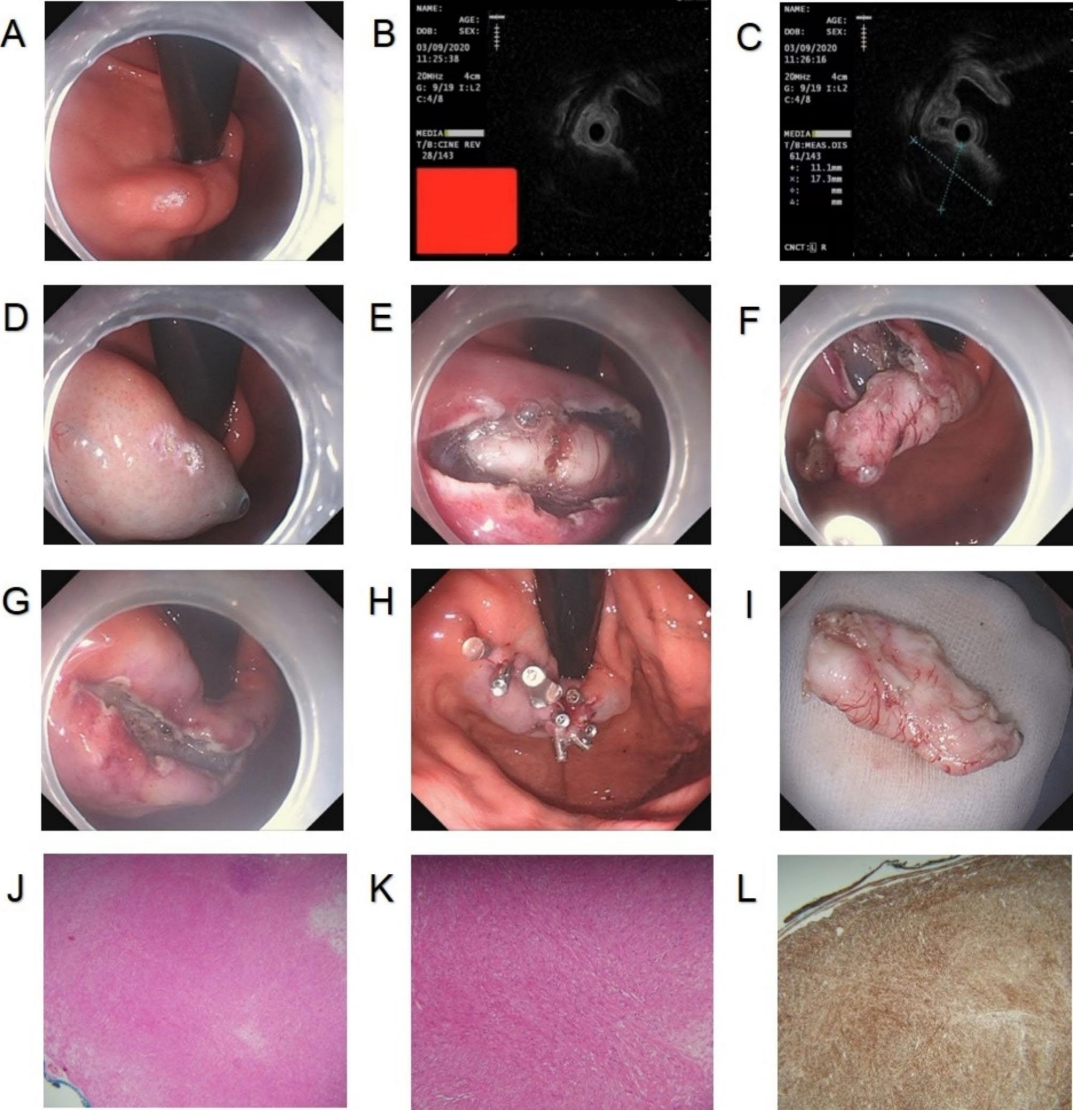
**A**. Submucosal tumor in the gastric fundic cardia under gastroscopy; **B**. A hypoechoic mass originating from the muscularis propria under EUS; **C**. Tumor section size under EUS: 1.73 cm*1.11 cm; **D**. Marking in the center of the tumor and submucosal injection; **E**. Longitudinal incision along the marks and exposure of the tumor; **F**. Dissection of the tumor along the peritoneum; **G**. Trauma after the dissection; **H**. Closure of trauma by titanium clip; **I**. Completely dissected tumor; **J-K**. Histopathological diagram of liomyoma; **L**. Positive Desmin expression in liomyoma tissues revealed by immunohistochemical staining. EUS, endoscopic ultrasound

Procedure: A mark point or a ring mark was made in the center of the lesion, followed by submucosal injection of the mixture (glycerol fructose + sodium glacial + indocyanine + epinephrine). Next, longitudinal incision of the muscularis mucosa and submucosa was conducted along the marks using an electric knife. Following separation of the submucosa and exposure of the lesion, the lesion was completely removed along its edge and base layer by layer (Fig. 3A-L).

##### Note

SMT originating from the muscularis mucosa and submucosa of the esophagus and stomach was located in shallow layers, and if the lesion was cut in the center after submucosal injection, the tumor and envelope were damaged easily. Hence, such SMT was excluded.

#### Endoscopic full-thickness resection (EFTR)

Adoption criteria: SMT originating from the muscularis propria of the stomach, with extraluminal growth.

**Fig. 4:**
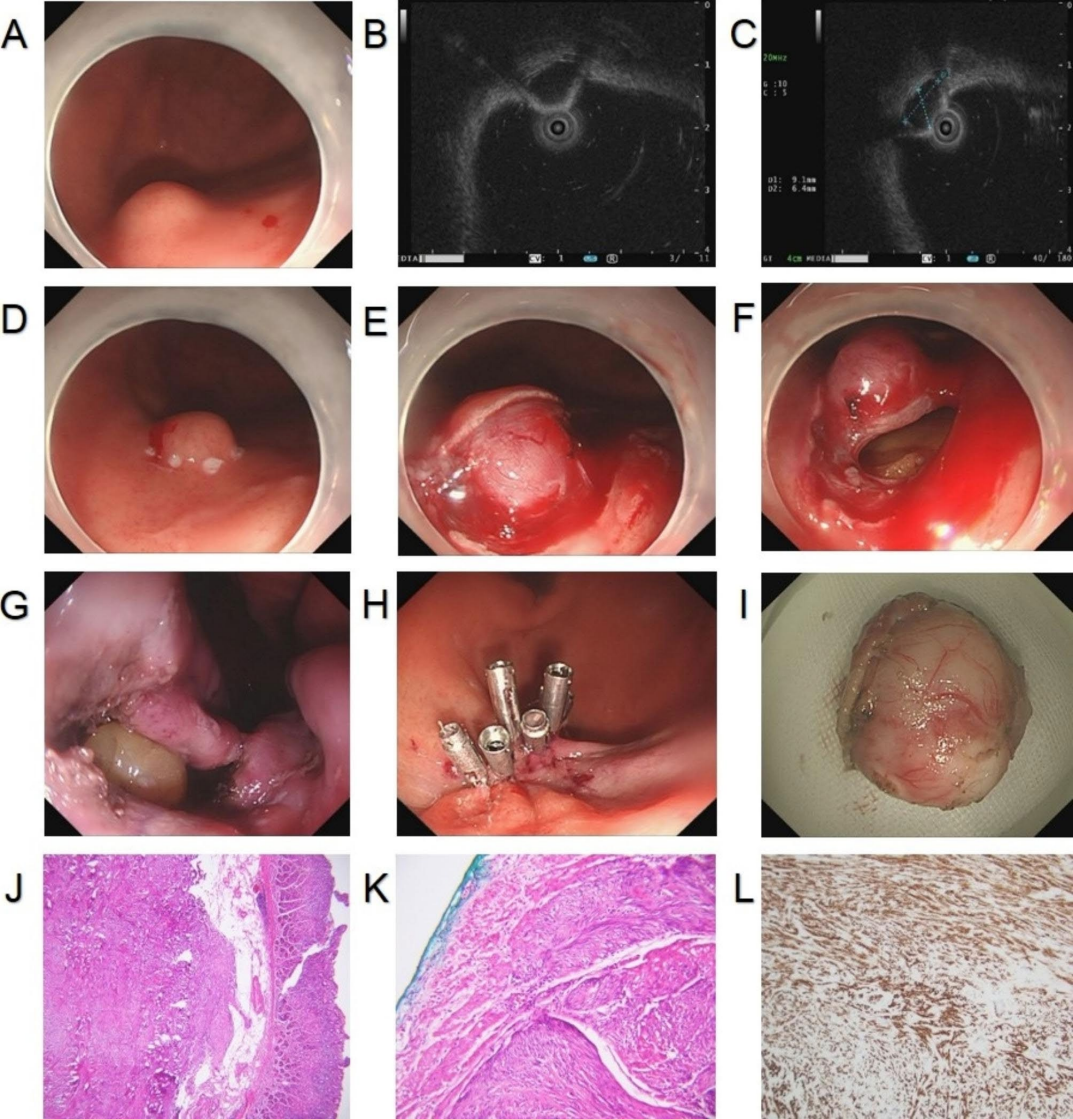
**A**. Submucosal tumor at the junction of the gastric fundic body under gastroscopy; **B**. Hypoechoic masses originating from the muscularis propria and with intraluminal growth under EUS; **C**. Tumor section size under EUS: 0.91 cm*0.64 cm; **D**. Marking of the edge of tumor; **E**. Exposure of the tumor after C-shaped incision along the marks; **F**. Incision of the plasma membrane for an “artificial perforation”; **G**. Trauma after the perforation; **H**. Closure of trauma by titanium clip; **I**. The complete dissection of the tumor; **J-L**. Histopathological diagram of mesenchymoma derived from the muscularis propria; CD117 was positively expressed in the tumor tissue. EUS, endoscopic ultrasound

Procedure: On the basis of ESD or ESE, the muscularis propria was exfoliated along the edge of the lesion to the plasma layer after the exposure of the lesion. Then, the plasma membrane was incised along the edge of the lesion to make an “artificial perforation” until the complete removal of the lesion. To avoid the resected tumor falling into the abdominal cavity, the lesion was fixed by means of a snare or foreign forceps and then removed [7] (Fig. 4A-L).

##### Note

The key to successful EFTR treatment is successful endoscopic repair of the perforation to avoid surgical repair and postoperative peritonitis [8]. Depending on the size of the trauma, endoscopic titanium clip closure, endoscopic purse-string suture, endoscopic omental repair and over-the-scope clip (OTSC) could be adopted for trauma closure. Intraoperatively, the abdomen of patients needed to be observed closely. Once pneumoperitoneum occurred, 5 ml of saline could be aspirated using a 10 ml syringe. After removing the injection core, a paracentesis was performed in the middle of the right abdomen to vent the air [7].

#### Submucosal tunneling endoscopic resection (STER)

Adoption criteria: SMT originating from muscularis propria of the esophagus and the cardia.

**Fig. 5:**
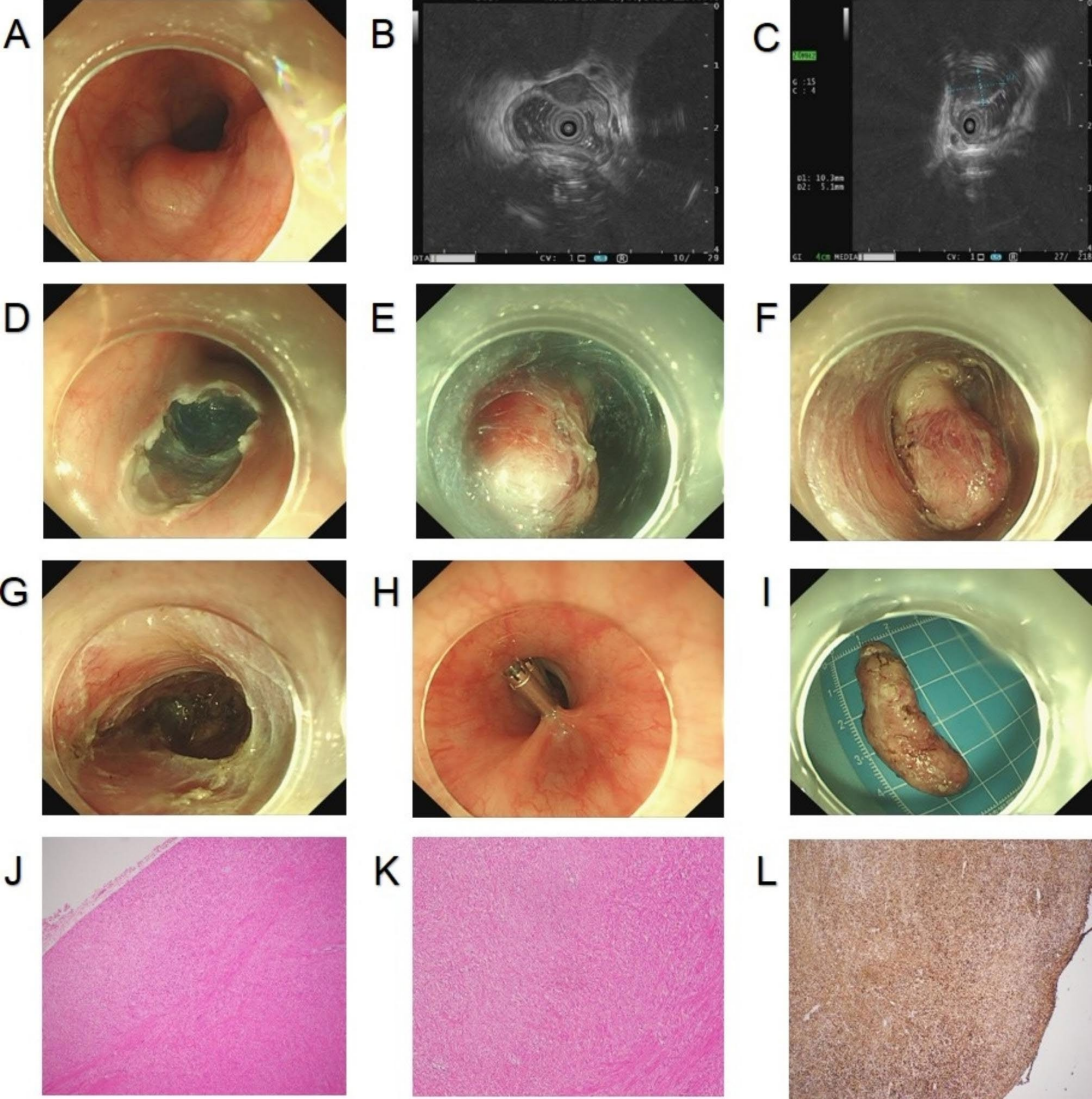
**A**. Submucosal tumor in the esophagus under gastroscopy; **B**. Hypoechoic masses originating from muscularis propria under EUS; **C**. Tumor section size under EUS: 1.03 cm*0.51 cm; **D**. Establishment of tunnel entrance; **E**. Discovery of the tumor in the tunnel; **F**. Dissection of the tumor layer by layer; **G**. Tunnel after removal of the tumor; **H**. Closure of the tunnel entrance by titanium clip; **I**. Complete dissection of the tumor; **J-L**. Histopathological diagram of liomyoma; Desmin was positively expressed in the tumor tissue. EUS, endoscopic ultrasound

Procedure: ① the mucosa was lifted first through a submucosal injection at 3–5 cm away from the lesion then incised longitudinally using an electric knife to establish the tunnel entrance (3–5 cm away from the pharynx). Notably, transverse incision was feasible for lesions in the cardia to establish the tunnel entrance; ② the tunnel was established 1–2 cm away from the anal side of the lesion; ③ within the tunnel, the lesion was completely removed; ④ after adequate hemostasis in the tunnel, the tunnel entrance was closed with a titanium clip (when the transverse incision is large, the purse-string suture could be adopted) (Fig. 5A-L).

#### Postoperative treatment

After surgery, the vital signs of patients were closely monitored, and symptoms such as haematemesis, melena, abdominal pain and chest pain. All patients were subjected to fasting for 24 h, all-day liquid diet for 1 day and half-day liquid diet for 3 days. Additionally, proton pump inhibitor was given intravenously for 3 days before being taken orally, with a total course of 1 month. If perforation or EFTR was performed, intravenous anti-infection treatment was given for 3 days simultaneously. After 1 month, gastroscopy was followed up.

#### Observed indicators

The surgery time, overall resection rate and intraoperative/postoperative complications of patients were recorded. The observed indicators included lesion size, distribution and pathological types; the correlation between lesion distribution and origin layer; the correlation between the origin layer and the choice of treatment; the consistency of the preoperative EUS diagnosis and postoperative pathological diagnosis.

## Results

### Basic postoperative characteristic of patients

Of 156 SMT patients, only 1 patient was referred to surgical treatment due to bleeding, the lesions of the rest of patients were removed successfully without postoperative complications, and the successful resection rate was 99.4%. There was no delayed bleeding or perforation after surgery. The surgery time was 8-180 min, with an average time of 40.07 min (Table 1).

**Table 1:** Distribution and pathological types of SMT in all patients

Pathological types	Location of lesions				
	Esophagus	Fundus or cardia of the fundus	Gastric body	Gastric sinus	Duodenum
Liomyoma	26	26	17	-	-
Mesenchymoma	-	42	17	-	-
Cyst	5	-	-	-	-
Granulosa cell Tumor	3	-	-	-	-
Lipomyoma	-	-	1	1	-
Heterotopic pancreas	1	-	1	3	2
Fibroma	-	-	5	-	-
Hemangioma	2	-	-	-	-
Lymphangioma	1	-	-	-	-
Schwannoma	-	-	1	-	-
Collagen nodule	-	-	-	1	-
Neuroendocrine tumor	-	-	-	-	1
Total	38	68	42	5	3

### Pathological types and origin layers of SMT under EUS in all patients

The tumor diameter of the selected 156 SMT patients was 0.3-4 cm, with an average diameter of 0.954 cm. Among them, 38 cases of lesions were located in the esophagus, 115 cases in the stomach (including 68 cases in the gastric fundus or fundic cardia, 42 cases in the gastric body, 5 cases in the gastric sinus), and 3 cases in the duodenum. Of 38 patients with esophageal SMT, the major pathological type was liomyoma, and the principal origin layers were the muscularis mucosa and the muscularis propria. Among 68 patients with SMT in the gastric fundus or fundic cardia, the dominant pathological type was mesenchymoma, second by liomyoma, and the origin layer was mainly the muscularis propria. Of 42 patients with SMT in the gastric body, the leading pathological types were mesenchymoma and liomyoma, and the major origin layer was the muscularis propria. Of 5 patients with SMT in the gastric sinus, the leading pathological type was heterotopic pancreas, and the main origin layer was muscularis submucosa. Among 3 patients with duodenal SMT, the major pathological types were heterotopic pancreas and neuroendocrine tumor, and the dominant origin layer was muscularis submucosa (Tables 1 and 2).

**Table 2:** The distribution and origin layer of SMT under EUS in all patients

Location	Cases	Muscularis mucosa	Muscularis submucosa	Muscularis propria
Esophagus	38	26	6	6
Fundus or cardia of the fundus	68	1	1	66
Gastric body	42	-	3	39
Gastric sinus	5	-	5	-
Duodenum	3	-	2	1

### Origin layers and selection of endoscopic treatments under EUS

Of 38 patients with esophageal SMT, ESD and EBL were mainly performed on those with the lesion originating from the muscularis mucosa and submucosa under EUS; and STER was mainly performed on those with the lesion originating from the muscularis propria. Among 115 patients with gastric SMT, ESE and EFTR were mainly performed on patients with the lesion originating from the muscularis mucosa and submucosa while ESE, ESD and EFTR were mainly performed on those with the lesion originating from the muscularis propria under EUS. Of 3 duodenal SMT patients, those who had a lesion derived from submucosa and muscularis propria under EUS mainly received SMT and ESD, respectively (Table 3).

**Table 3:** Origin layers under EUS and the selection of endoscopic treatments

Location	Origin layer	Cases	EBL	ESD	ESE	EFTR	STER
Esophagus	Muscularis mucosa	26	4	22	-	-	-
	Muscularis submucosa	6	3	3	-	-	-
	Muscularis propria	6	1	-	-	-	5
Stomach	Muscularis mucosa	1	-	1	-	-	-
	Muscularis submucosa	9	-	9	-	-	-
	Muscularis propria	105	3		72	30	-
Duodenum	Muscularis submucosa	2	-	2	-	-	-
	Muscularis propria	1	-		1	-	-

EBL: Endoscope band ligation; ESD: Endoscopic submucosal dissection; ESE: Endoscopic submucosal excavation; EFTR: Endoscopic full-thickness resection; STER: Submucosal tunneling endoscopic resection (STER)

### Analysis of preoperative EUS diagnosis and postoperative pathological diagnosis results

Through preoperative EUS combined with the origin layer and echogenicity of lesions in all patients, the nature of lesions were determined initially. After surgery, all specimens were subjected to pathological examination. The consistency between the EUS diagnosis and the postoperative pathological nature could be observed in 121 cases of patients (consistency rate: 84.6%) (Table 4).

**Table 4:** Analysis of preoperative EUS diagnosis and postoperative pathological diagnosis results

Diagnostic methods	Cases	Liomyoma	Mesenchymoma	Cyst	Lipomyoma	Heterotopic pancreas	Neuroendocrine tumor
Postoperative pathological diagnosis	143	69	59	5	2	7	1
Preoperative EUS diagnosis	121	49	59	5	2	5	1

## Discussion

Based on the popularity of health awareness and gastroscopy, the incidence of SMT has increased year by year [9]. In recent years, the rapid development of endoscopic techniques and equipment has witnessed gastroscopic resection surgery as the main treatment for SMT [10]. There are various endoscopic treatments, and how to precisely make a selection is important. In this study, a total of 156 SMT patients were retrospectively analyzed. Briefly, most of lesions occurred in the esophagus, gastric fundus or fundic cardia and gastric body; as for the distribution of pathological types, liomyoma was most common in the esophagus, liomyoma and mesenchymoma in the fundic cardia and gastric body, and heterotopic pancreas in the gastric sinus. Through EUS, SMT was discovered to mostly originate from the mucosal myocardium and muscularis propria in the esophagus, the muscularis propria in the fundus cardia and gastric body, and the submucosa in the gastric sinus, which coincided with the common pathological types of lesions in each site.

EUS can not only initially determine the pathological type of lesions with the help of the origin layer and echogenicity of lesions, but also guide the precise application of endoscopic treatments through taking the size and growth pattern of lesions into account [11]. A retrospective study compared the accuracy of EUS and CT in diagnosing SMT. The analysis results revealed the diagnostic rate of EUS and CT for mesenchymoma (83.9% vs. 74.2%), liomyoma (37.5% vs. 0), and heterotopic pancreas (57.1% vs. 14.3%); the diagnostic rate of EUS was higher than that of CT [12]. However, EUS also has some limitations. On the one hand, it can only display a certain section of the tumor, while the origin layer, shape and size of the tumor displayed by this section may not be consistent with other sections. On the other hand, some common SMTs such as liomyoma, mesenchymoma, cyst, lipomyoma, heterotopic pancreas, and euroendocrine tumor can be easily diagnosed before surgery through combining the characteristic manifestations under white light endoscope and EUS. However, the specificity of EUS is low for rare tumors such as granulosa cell tumor, hemangioma, lymphangioma, and schwannoma. EUS-guided fine-needle aspiration (EUS-FNA) is then required, because imaging of layer IV tumors is very difficult to identify, even with contrast echo. However, EUS-FNA is not easily performed for subepithelial lesions [13]. Moreover, EUS diagnosis requires operating skills and experience, and only skilled doctors and experienced experts can achieve a high diagnostic accuracy of EUS. It is worth noting that histopathological examination is always the gold standard for the diagnosis of SMT. Hence, the higher the diagnostic accuracy of EUS before surgery, the more favorable it is for doctors to choose treatment methods and achieve the therapeutic purpose of complete resection. The retrospective analysis of SMT patients in our endoscopy center showed that conventional ESD was applied to treat lesions originating from the muscularis mucosa and submucosa of the esophagus with a diameter of ≤ 0.8 cm in the past. In nearly half a year, only applying EBL alone could ensure complete excision of the lesions, shorten the operation time, and save medical costs. However, conventional ESD was responsible for lesions originating from the muscularis mucosa and submucosa of the esophagus with a diameter of > 0.8 cm. As for lesions originating from the muscularis propria of the esophagus that were in line with endoscopic indications, STER was used to avoid postoperative complications and shorten the hospital stay by ensuring the integrity of the mucosal layer of the esophagus, regardless of the size of the lesion.

In our endoscopic center, EBL or traditional ESD was frequently used for SMT originating from the gastric muscularis mucosa, submucosa and propria with a diameter of ≤ 0.8 cm. Briefly, ESD was employed for SMT originating from the gastric muscularis mucosa and submucosa, regardless of the size of the lesion. EBL was adopted for SMT originating from the gastric muscularis mucosa, submucosa and propria and with a diameter of ≤ 0.8 cm. Notably, ESE is also usually selected for the treatment of SMT originating from propria. In comparison with ESE, EBL has a short operative time and low operative cost. Normally, the lesion is too small to be detected after submucosal injection and incision using ESE. However, EBL method is prone to perforation following ligation of lesions in the muscularis propria of the stomach, especially in the thinner propria of the fundus, and further leads to the fall of the rubber ring and the mass into the abdominal cavity [14]. Hence, the snare should be placed under the rubber ring as much as possible during ligation. With regard to intraoperative bleeding, the bleeding vessels can be pre-treated or intuitively dealt with because the surgical field of view is intuitive throughout the ESE procedure; whereas in EBL, the vessels cannot be pre-treated before electroresection and are prone to bleeding after electroresection [15]. The trauma will appear unclear due to the bleeding, and it is particularly challenging to locate and treat the bleeding vessels. Therefore, it is highlighted that an appropriate frequency of electrocoagulation should be chosen during electroresection, and that the snare cannot be collected too quickly. Overall, both surgical methods have their own advantages and disadvantages, and the appropriate choice between them depends on the preoperative assessment of the lesion, as well as the own situation of the patient.

For SMT originating from gastric muscularis mucosa, submucosa and muscularis propria with a diameter of > 0.8 cm and intraluminal growth, conventional ESE was adopted (the mass was removed after making a longitudinal incision along the center of the lesion or a circumferential incision of the mucosa and submucosa). Making a comparison between the two incision methods, longitudinal incision contributes more to finding small lesions, and circumferential incision is more suitable for finding large lesions. Zhang et al. [16] believed that a linear incision or cross incision in the center of the tumor is suitable for SMT with a maximum diameter of 2 cm or less, and a circumferential incision along the side of the tumor for that with a diameter more than 2 cm.

For SMT originating from the muscularis propria of the stomach, especially with extraluminal growth, the plasma membrane layer can be cut to make an “artificial perforation” to ensure complete excision of the lesion, which is called EFTR [17]. By the way, excellent suturing abilities of endoscopic physicians are necessary for the application of EFTR. If necessary, intraoperative laparoscopic venting needs to be performed. Additionally, both timely postoperative anti-infective treatment and close monitoring of the disease can’t be ignored [18].

This study was a retrospective research with a small sample size. Therefore, a multi-center and large-sample research requires to be carried out to further verify the results of this study in the future.

## Conclusion

Through reviewing 156 patients in our endoscopy center, we have discovered that clarifying the origin layer, size, growth pattern and pathological nature of lesions using preoperative EUS can not only guide precise selections of the surgical modality but also ensure the surgical effect and reduce complications. Therefore, preoperative EUS deserves a clinical promotion. In addition, we believe that this study has certain reference significance for endoscopists, especially young endoscopists.

## Data Availability

The datasets used and/or analysed during the current study available from the corresponding author on reasonable request.
